# Productive replication of nephropathogenic infectious bronchitis virus in peripheral blood monocytic cells, a strategy for viral dissemination and kidney infection in chickens

**DOI:** 10.1186/s13567-016-0354-9

**Published:** 2016-07-13

**Authors:** Vishwanatha R. A. P. Reddy, Ivan Trus, Lowiese M. B. Desmarets, Yewei Li, Sebastiaan Theuns, Hans J. Nauwynck

**Affiliations:** Laboratory of Virology, Department of Virology, Parasitology and Immunology, Faculty of Veterinary Medicine, Ghent University, Salisburylaan 133, 9820 Merelbeke, Belgium

## Abstract

In the present study, the replication kinetics of nephropathogenic (B1648) and respiratory (Massachusetts-M41) IBV strains were compared in vitro in respiratory mucosa explants and blood monocytes (KUL01^+^ cells), and in vivo in chickens to understand why some IBV strains have a kidney tropism. B1648 was replicating somewhat better than M41 in the epithelium of the respiratory mucosa explants and used more KUL01^+^ cells to penetrate the deeper layers of the respiratory tract. B1648 was productively replicating in KUL01^+^ monocytic cells in contrast with M41. In B1648 inoculated animals, 10^2.7–6.8^ viral RNA copies/100 mg were detected in tracheal secretions at 2, 4, 6, 8, 10 and 12 days post inoculation (dpi), 10^2.4–4.5^ viral RNA copies/mL in plasma at 2, 4, 6, 8, 10 and 12 dpi and 10^1.8–4.4^ viral RNA copies/10^6^ mononuclear cells in blood at 2, 4, 6 and 8 dpi. In M41 inoculated animals, 10^2.6–7.0^ viral RNA copies/100 mg were detected in tracheal secretions at 2, 4, 6, 8 and 10 dpi, but viral RNA was not demonstrated in plasma and mononuclear cells (except in one chicken at 6 dpi). Infectious virus was detected only in plasma and mononuclear cells of the B1648 group. At euthanasia (12 dpi), viral RNA and antigen positive cells were detected in lungs, liver, spleen and kidneys of only the B1648 group and in tracheas of both the B1648 and M41 group. In conclusion, only B1648 can easily disseminate to internal organs via a cell-free and -associated viremia with KUL01^+^ cells as important carrier cells.

## Introduction

Avian infectious bronchitis virus (IBV) causes mild to acute respiratory disease in chickens, characterized by coughing, sneezing, tracheal rales and dyspnea [1]. IBV belongs to the order of the *Nidovirales*, family *Coronaviridae*, subfamily *Coronavirinae* and genus *Gammacoronavirus* [2]. Worldwide, IBV causes huge economic losses in both broilers and layers. IBV has a tropism not only for the epithelium of the respiratory tract but also for the epithelium of kidneys, oviduct, gastrointestinal tract (oesophagus, proventriculus, duodenum, jejunum, bursa of Fabricius, caecal tonsils, rectum and cloaca) and testes [3, 4]. IBV is clinically associated with poor performance of birds, reduced egg production and quality, as well as increased predisposition to other secondary bacterial infection [5]. IBV is highly contagious. Currently, multiple serotypes of IBV exist, and new variants emerge due to frequent point mutations and recombination events in the viral genome [4]. Vaccination failure is very common against IBV due to poor or no cross-protection between different IBV serotypes.

The first IBV was isolated from birds showing respiratory problems in the United States in 1931 [6]. In the early 1950s, the well-known respiratory Massachusetts type of IBV (Mass) was isolated in the United States. In subsequent years, Mass-type (prototype: M41) strains have been identified worldwide, and many variants emerged. Some IBV strains were called nephropathogenic because the initial respiratory infection was followed by severe kidney infection. Important clinical signs of nephropathogenic IBV strains include increased water consumption, low body weight gain, watery droppings and significant mortality. Necropsy of birds that died during a nephropathogenic infection reveals enlarged and pale kidneys with urates in the collecting tubules [7]. In the 1960s, the first nephropathogenic IBV strains were reported in the US and Australia, and later worldwide. In the last 15 years, nephropathogenic IBV strains have been emerging as most prevalent IBV strains in commercial poultry [8–12]. The B1648 strain is a Belgian reference nephropathogenic IBV serotype, that was responsible for large outbreaks of kidney disease in broiler farms in Belgium, The Netherlands and Northern France, and was first isolated in 1984 [7, 13–15].

In September 2012, a novel coronavirus emerged in humans, designated Middle East respiratory syndrome coronavirus (MERS-CoV). MERS-CoV has a higher mortality rate (>35%) than another well-known coronavirus, the severe acute respiratory syndrome coronavirus (SARS-CoV) (9.6%). The MERS-CoV infected patients usually end up with a severe pneumonia complicated with kidney failure. The severity of MERS-CoV infections in humans, caused by its extra-pulmonary infection of kidneys have prompted us to question why this virus has a strong tropism for the kidneys. The same question has been raised for the kidney tropism of certain IBV strains, for the past 25 years [7, 13–15]. Hence, in the present study, we aimed to explore the tissue tropism characteristics of IBV nephropathogenic (B1648) and respiratory (M41) strains in chickens. To this end, replication kinetics of IBV B1648 and M41 were evaluated in vitro in tracheal mucosa explants and blood monocytes by a reproducible quantitative analysis system using confocal microscopy [16–18]. A new 5′ RT-qPCR was validated and used for comparing in vivo the viral replication kinetics in the respiratory tract and dissemination in blood of IBV B1648 and M41 [19]. Elucidating the tissue tropism mechanisms of B1648 and M41 is important to plan better prevention strategies for emerging highly nephropathogenic IBV infections.

## Materials and methods

### IBV B1648 and M41 replication characteristics in tracheal mucosa explants and peripheral blood monocytes

#### Viruses

The virulent nephropathogenic IBV B1648 and the respiratory prototype M41 were used in this study. B1648 is a Belgian field isolate obtained in 1984 and described previously [13, 15, 20]. M41 with unknown passage history was obtained from the avian pathology laboratory, Ghent University [21]. Virus was propagated in specific pathogen free (SPF) 10 days old embryonated chicken eggs. From both B1648 and M41, a second passage was produced and utilized in the present experiment. The allantoic fluids were harvested 48 h after infection, clarified by low-speed centrifugation and stored at −70 °C. Virus titration was performed in 10 days old chicken embryos by inoculation of 10-fold dilutions via allantoic route and expressed as 50% egg infectious dose per mL (EID_50_/mL).

#### Virus titration

Intracellular and extracellular virus titers were determined by an EID_50_ assay using embryonated chicken eggs. Embryonated chicken eggs were obtained from an SPF flock and used when the embryos were 10 days old. Ten-fold dilutions of virus were prepared in PBS (1 × Dulbecco’s phosphate buffered saline (DPBS) with 0.9 mM CaCl_2_, 0.5 mM MgCl_2_ × 6H_2_O and 20 mg/L phenol red) [4] and 100 μL of the dilutions were inoculated into the allantoic cavity of eggs. Eggs were incubated at 37 °C for 7 days. The embryos were collected and examined for the presence of embryo dwarfing and curling, which is characteristic for an IBV infection. Based on the observations, end points were calculated [7, 22]. Virus titers were assessed by a fifty percent end-point, according to the method of Reed and Muench, and expressed as EID_50_/mL.

#### Isolation and cultivation of chicken tracheal mucosa explants

This study was performed in agreement with the guidelines of the local Ethical and Animal Welfare Committee of the Faculty of Veterinary Medicine and Bio-Science Engineering of Ghent University. The chicken tracheal mucosa explants were prepared as described previously [23]. Briefly, three eight-week-old specific-pathogen free (SPF) chickens were euthanized by intravenous injection of sodium pentobarbital (100 mg/kg) in the brachial wing vein. Tracheas were collected and carefully split into two equal halves with surgical blades (Swann–Morton). Tracheal mucosal explants covering a total area of 10 mm^2^ were made and placed on top of fine-meshed gauzes in six-well culture plates (Nunc), epithelium upwards. The explants were maintained in serum-free medium [50% Ham’s F12 (Gibco)/50% DMEM (Gibco)] supplemented with 100 U/mL penicillin (Continental Pharma), 0.1 mg/mL streptomycin (Certa), 1 μg/mL gentamycin (Gibco) and 25 mM HEPES (Gibco). The epithelium of the explants was slightly immersed in the fluid to achieve an air liquid interface. Explants were maintained up to 96 h at 37 °C and 5% CO_2_.

#### Isolation of blood monocytes

Five mL blood was collected on heparin (15 U/mL) (Leo) from the brachial wing vein of three chickens. Monocytes were isolated from chicken peripheral blood mononuclear cells (PBMC) by Ficoll-paque gradient centrifugation as described by the manufacturer (Pharmacia Biotech AB). Mononuclear cells were resuspended in RPMI-1640 (Gibco) medium containing 10% fetal calf serum (FCS, Gibco), 100 U/mL penicillin (Continental Pharma), 0.1 mg/mL streptomycin (Certa), 1 μg/mL gentamycin (Gibco), and 1% non-essential amino acids (Gibco). Afterwards, 2 × 10^6^ cells/mL were seeded in a 24-well plate (Nunc) and cultivated at 37 °C with 5% CO_2_. Non-adherent cells were removed by washing two times with RPMI-1640 medium at 2 and 24 h after seeding. The adherent cell population consisted of 80.2 ± 7.3% of monocytes, based on the fluorescent staining with the monocyte/macrophage marker KUL01 [24]. KUL01^+^ cells were cultured up to 96 h at 37 °C and 5% CO_2_. All experiments were performed in triplicate.

#### IBV inoculation and sample collection of tracheal mucosa explants and monocytes

Tracheas of three chickens were used. Explants were inoculated with IBV B1648 and M41 at 24 h of cultivation. For the inoculation, explants were taken from their gauze and placed at the bottom of a 24-well plate with the epithelial surface upwards. Explants were washed twice with warm serum-free medium and inoculated with 1 mL of a virus stock containing 10^7.0^ EID_50_ (1 h, 37 °C, 5% CO_2_). In in vitro tracheal mucosa experiments, the local mucus layer and other innate defense barriers reduce infectivity of the mucosa. Therefore, the mucosal explants were washed before inoculation to remove mucus and other innate defense barriers and high virus titers were used (10^7^ EID_50_/mL) in order to have sufficient virus to reach the epithelial cells. In addition, the main aim of this study was to evaluate replication kinetics at early time points, before the viability of the mucosal explants goes down, i.e. 96 h of cultivation and 72 h after virus inoculation. To meet the above expectations, the use of a high virus titer for the inoculation of in vitro mucosal explants is a general standardized protocol in our laboratory [17, 25, 26]. After 1 h of virus incubation, the inoculated explants were washed three times with warm medium and placed back on the gauze. Explants were collected at 0, 6, 12, 24, 48 and 72 h post-inoculation (hpi), embedded in Methocel^®^ (Fluka) and frozen at −70 °C.

Monocytes were inoculated with IBV B1648 and M41 at a multiplicity of infection (m.o.i.) of 5. To maintain uniformity and consistency, the virus titer of inoculum, incubation time, washing steps and sample collection time points should be similar in TOC and monocyte cultures under in vitro conditions. Therefore, the same virus titer was used to inoculate in vitro cultured monocytes. After 1 h of virus incubation (37 °C, 5% CO_2_), cells were washed three times with warm RPMI-1640 medium and further incubated in medium. Cells and supernatant were collected at 0, 6, 12, 24, 48 and 72 hpi for viral antigen quantification and virus titration. The cells were removed from the well by scraping and added to the pellet for determination of intracellular virus titers. Virus was released from the cells by 2 freeze–thaw cycles. The supernatants were used for determination of extracellular virus titers. The samples were stored at −70 °C until virus titration on embryonated eggs.

#### Identification and quantification of IBV infected cells in the tracheal mucosa explants and monocytes

A double immunofluorescence staining was performed to identify and quantify individual IBV-positive cells. At 0, 6, 12, 24, 48 and 72 hpi, 15 consecutive cryosections of 10 μm were made from the frozen tracheal explants and fixed (4% paraformaldehyde for 10 min and 0.1% Triton^®^ X-100 for 2 min). The cryosections were first stained for monocytes/macrophages (KUL01 marker), and next for IBV antigens. The cryosections were incubated (1 h, 37 °C) with mouse monoclonal anti-chicken monocyte/macrophage (KUL01) antibodies (Southern Biotech) (1:250 in PBS containing 10% negative goat serum) and washed three times after incubation. Cryosections were then incubated (1 h, 37 °C) with Texas Red-labelled goat anti-mouse IgG_1_ antibodies (Molecular Probes) (1:250 in PBS containing 10% negative goat serum and 10% negative chicken serum). Subsequently, after washing, the sections were incubated (1 h, 37 °C) with a polyvalent IBV hyperimmune serum conjugated with FITC [14, 20] (1:20 in PBS for B1648; 1:7 in PBS for M41). Finally, after washing, the sections were incubated [10 min, room temperature (RT)] with Hoechst 33342 (1:100 in PBS), washed and mounted with glycerin-DABCO (Janssen Chimica).

Based on cross serum neutralization tests, B1648 is distantly related to respiratory IBV strains. FITC-conjugated antibodies were prepared from a polyclonal hyperimmune serum against B1648 strain, and their reactivity against B1648 infected cells was quite different from the reactivity against M41 infected cells [14, 20]. As a result, the concentration of FITC-conjugated polyclonal anti-B1648 antibodies that is needed to detect IBV-positive cells was higher for M41 than for B1648 [21]. The IBV-positive cell quantification done with FITC-conjugated polyclonal anti-B1648 antibodies was confirmed by monoclonal antibodies directed against the nucleocapsid protein [27]. The number of virus-infected cells was determined in 15 consecutive cryosections in both the epithelium and lamina propria of the tracheal mucosa explants. Further, at each time point, the number of infected KUL01^+^ cells was calculated both in the epithelium and lamina propria.

Monocytes were fixed in 4% paraformaldehyde for 10 min and 0.1% Triton^®^ X-100 for 2 min at 0, 6, 12, 24, 48 and 72 hpi. IBV-positive cells in monocytes were visualized using the same technique as for the cryosections. The percentage of B1648 and M41-positive cells was determined for the KUL01^+^ cells (colocalization). A reproducible quantitative analysis system was followed for the elucidation of IBV replication in the tracheal mucosal explants and blood monocytes/macrophages (KUL01^+^ cells) by using a confocal microscopy (Leica TCS SPE confocal microscope) [16–18].

### Replication kinetics of IBV B1648 and M41 in chickens

#### Experimental design

The experimental setup is presented in Table 1. Nine 3-week-old SPF White Leghorn chickens were individually tagged and 3 chickens per group were housed in separate negative pressure (150 pa) isolation units (IM 1500, Montair). Before the start of the experiment, an acclimatization period of two-weeks was respected. Drinking water and food were provided ad libitum. The experimental design was evaluated and approved by the Ethical and Animal Welfare Committee of the Faculty of Veterinary Medicine of Ghent University (EC 2014/160).

**Table 1:** **Experimental design of in vivo IBV (B1648/M41) infection study**

Group	Number of animals	Time of inoculation (days post inoculation)			
Virus strain		Observation of clinical signs	Tracheal swab collection	Blood collection (plasma and PBMC^a^)	Euthanasia
B1648	3	−3 to 12	0, 2, 4, 6, 8, 10, 12	0, 2, 4, 6, 8, 10, 12	12
M41	3	−3 to 12	0, 2, 4, 6, 8, 10, 12	0, 2, 4, 6, 8, 10, 12	12
PBS control	3	−3 to 12	0, 2, 4, 6, 8, 10, 12	0, 2, 4, 6, 8, 10, 12	12

^a^Peripheral blood mononuclear cells.

#### Virus inoculation

Three 5-week-old chickens were inoculated with the virulent nephropathogenic B1648 strain or respiratory M41 via intratracheal (200 μL), nasal (50 μL each nostril) and ocular routes (50 μL each eye) with 10^3^ EID_50_/400 μL. The main aim of our in vivo study was to study the replication kinetics of IBV in the respiratory mucosa and its spread via blood to internal organs (0 to 12 dpi). To conduct this in vivo study successfully, mortality should not be induced. Based on our experience of past experiments and available literature, we have selected 10^3^ EID_50_/400 μL to keep the chance of mortality at a minimal level [20, 28, 29]. The control chickens were mock inoculated with PBS and served as a negative control group.

#### Clinical signs and water consumption

Clinical signs, such as depression, huddling together, ruffled feathers, sneezing, coughing, tracheal rales and dyspnoea were recorded daily from −3 until 12 days post inoculation (dpi). Waterers were regularly weighed in each group to calculate water consumption per bird.

#### Post mortem findings

At 12 dpi, the chickens from the three groups were humanely euthanized. During necropsy, all tissues were examined macroscopically. After macroscopical examination, two samples were collected from the following tissues: trachea, lungs, liver, spleen and kidneys. The first tissue sample was stored at −70 °C for viral RNA quantification by RT-qPCR. The second tissue sample was embedded in Methocel^®^ (Fluka) and was snap frozen for immunofluorescence staining.

#### Standardization of SYBR green-based RT-qPCR assay for ORF 1a gene

To avoid the detection of subgenomic mRNAs, the real-time 5′ RT-qPCR primers were designed by targeting the highly conserved region of open reading frame (ORF) 1a of B1648 strain (Table 2). Then, a single step SYBR green-based 5′ RT-qPCR assay was developed for ORF1a gene.

**Table 2:** **Primers used for real-time RT-PCR of ORF 1a**

Primer name	Gene	Primer sequence	Amplicon Size
cDNA_fw	ORF 1a	5′-GGT GTT AGG CTT ATA GTT CCT CAG-3′	254 nt
cDNA_rv	ORF 1a	5′-TAA ACA TTA GGG TTG ACA CCA GT-3′	
T7_fw	ORF 1a	5′-*TAA TAC GAC TCA CTA TAG GG*G GTG TTA GGC TTA TAG TTC CTC AG-3′	
qPCR_fw	ORF 1a	5′-GCT ATT GTA GAG GTA GTG TAT GTG AG-3′	176 nt
qPCR_rv	ORF 1a	5′-AGG GTT GAC ACC AGT AAA GAA T-3′	

#### Primer design and in silico validation

Multiple sequence alignments were performed with the relevant IBV sequences of GenBank in the MEGA software version 5.2.2. The real-time RT-qPCR primers were designed using the online web tool Primer 3 plus. The sequences of the primers for real-time RT-qPCR targeting ORF 1a and amplicon size are listed in Table 2. Primer-specificity was assessed in silico by use of the Basic Local Alignment Search Tool (BLAST) in public databases and by aligning these primers to *ORF 1a* genes of pan IBV strains of the worldwide. The risk of primer-dimer formation, and the presence of hairpins at the annealing site were analyzed using OligoAnalyzer 3.1 (Integrated DNA Technologies) with a correction for ion concentrations set at 50 mM for Na^+^ and 3 mM for Mg^2+^. All primers used in this study were synthesized by Integrated DNA Technologies and purified by standard desalting.

#### Preparation of RNA standards for absolute quantification

Standard RNA for absolute quantification was prepared from B1648. Viral RNA of the B1648 was extracted using the QIAamp Viral RNA Mini Kit (Qiagen). A synthetic fragment containing the amplicon size of 254 nt of *ORF 1a* gene was generated in vitro to prepare the standard RNA for absolute quantification. The RNA was reverse-transcribed into cDNA with the QIAGEN OneStep RT-PCR Kit (Qiagen), using primer set ORF 1a cDNA_fw/cDNA_rv (Table 2). ORF 1a cDNA_fw primer was modified to T7_fw with a T7 promoter sequence at its 5′ end by Herculase II fusion DNA polymerase (Agilent Technologies Inc., Santa Clara, CA, USA). The T7 promoter sequence incorporation was necessary for subsequent in vitro transcription (Table 2). A 50 μL OneStep RT-PCR (Qiagen) reaction mixture consisting 10 μL OneStep RT-PCR buffer (Qiagen), 2 μL dNTP (10 mM) mix, 3 μL forward primer (10 μM), 3 μL reverse primer (10 μM), 2 μL OneStep RT-PCR enzyme mix, 20 μL nuclease free water and 10 μL RNA. To generate the cDNA fragment, a reverese transcription step of 30 min at 50 °C and an enzyme activation step of 15 min at 95 °C were followed by 35 cycles each denaturation 30 s at 94 °C, annealing 30 s at 48 °C and extension 1 min at 72 °C. A final extension step was performed for 10 min at 72 °C and stored at 4 °C until further processing. Then, the cDNA fragment was analyzed by 2% agarose gel electrophoresis, and fragments with the correct length were excised and purified from gel using the Nucleospin Gel and PCR-Clean up kit (Macherey–Nagel). The cDNA was either used directly for in vitro transcription or stored at −70 °C. In vitro transcription of RNA was performed by incubation for 1 h at 37 °C with 10× transcription buffer, 500 μM rNTPs and 20 U T7 RNA Polymerase-Plus Enzyme Mix (Applied Biosystems). Template DNA was removed by treatment with enzyme 2U DNase I (Sigma Aldrich) for 15 min at 37 °C. Then, enzyme DNase I inactivation was done for 10 min at 75 °C. Finally, the in vitro generated RNA was purified using the RNeasy Mini Kit (Qiagen) and the amount of RNA was determined using the Nanodrop 2000 Spectrophotometer (Thermo Scientific). RNA standards were stored in single-use aliquots of 20 μL (8 ng/μL, 5.27 × 10^10^ copies/μL) volume at −70 °C. Ten microliters of RNA was used for preparation of a standard curve, and the remainder (6 μL) was used to determine the RNA concentration with the Nanodrop 2000 Spectrophotometer. The ENDMEMO online web tool was used to calculate the number of RNA copies per microliter.

#### SYBR green based one step RT-qPCR protocol

In order to generate a standard curve, the concentration of RNA was measured as described above, and further serially ten-fold diluted in nuclease free water (Gibco). RT-qPCR reaction mixtures (20 μL) consisted of 10 μL Precision OneStep qRT-PCR Mastermix with SYBR Green and ROX (Primer Design), 200 nM (0.4 μL/reaction) of each primer (qPCR_fw and qPCR_rv), 6.2 μL nuclease free water and 3 μL of standard RNA template or H_2_O. Reaction mixtures were loaded in MicroAmp Optical 96-well reaction plates (Applied Biosystems), sealed with MicroAmp Optical Adhesive Films (Applied Biosystems), and experiments were performed in a StepOnePlus apparatus (Applied Biosystems). A reverse transcription step of 10 min at 55 °C and an enzyme activation step at 95 °C for 8 min were followed by 40 cycles, each 10 s at 95 °C and 60 s at 58 °C. Afterwards, a first-derivative melting curve analysis was performed by heating the mixture to 95 °C for 15 s, then cooling to 60 °C for 1 min, and heating back to 95 °C at 0.3 °C increments. Results were analyzed using the StepOnePlus Software version 2.2. The baseline was set automatically, and the threshold was placed manually in the exponential phase of the amplification reaction. Melt curve analysis and agarose gel electrophoresis were performed to assess specificity of the reactions. Amplification efficiency was determined by running a standard curve over a linear dynamic range (LDR) from 7 log_10_ copies/reaction to 1 log_10_ copies/reaction in 1:10 dilution steps. Each dilution point of the standard curve was analyzed in triplicate and also three non-template control reactions (nuclease free water) were included in each experiment.

#### Collection of tracheal secretions, plasma and PBMCs

Tracheal secretions and 2 mL of blood [with EDTA (VWR)] were collected from each chicken at 0, 2, 4, 6, 8, 10 and 12 dpi. Immediately after collection, tracheal secretions were immersed in transport medium containing PBS [1 × Dulbecco’s PBS (DPBS) with 0.9 mM CaCl_2_, 0.5 mM MgCl_2_ × 6H_2_O and 20 mg/L phenol red] supplemented with 10% FCS, 1000 U/mL penicillin, 1 mg/mL streptomycin and 0.5 mg/mL kanamycin. Plain swabs were weighed before and after swabbing, and viral RNA copies were calculated per 100 mg of secretions. Plasma was harvested from blood after centrifugation (300 *g*, 10 min) and the remaining pellet was used for isolation of PBMCs (mononuclear cells). Mononuclear cells were isolated by Ficoll-paque gradient centrifugation as described above (section “IBV B1648 and M41 replication characteristics in tracheal mucosa explants and peripheral blood monocytes”). Infection in mononuclear cells was evaluated by three methods. With the first method, 200 000 mononuclear cells were used to make cytospins and to perform immunostainings (protocol was described in section “IBV B1648 and M41 replication characteristics in tracheal mucosa explants and peripheral blood monocytes”), to quantitate the number of viral antigen positive cells. With the second method and third method, 10^6^ mononuclear cells were lysed by one freeze–thaw cycle to determine the number of viral RNA copies by RT-qPCR, and to confirm the presence of infectious virus by virus isolation, respectively. Plasma and 10^6^ mononuclear cells were stored at −70 °C until use.

#### RT-qPCR in tracheal secretions, plasma and mononuclear cells

Viral RNA was extracted from tracheal secretions, plasma and mononuclear cells using the QIAamp Viral RNA Mini Kit (Qiagen). Three μL of RNA was used per RT-qPCR reaction. Viral RNA extracts from tracheal secretions, plasma and mononuclear cells were analyzed in duplicate RT-qPCR reactions as described above.

#### Virus isolation in plasma and mononuclear cells

Virus isolation from the plasma and 10^6^ mononuclear cells samples were performed on 10 days old embryonated SPF eggs, to confirm the presence of infectious virus (protocol described in section “IBV B1648 and M41 replication characteristics in tracheal mucosa explants and peripheral blood monocytes”). If embryo dwarfing and curling lesions were not observed after the first inoculation, up to five blind passages of the samples were performed.

#### RT-qPCR and immunofluorescence staining of tissues

RNA was extracted from tissues using the QIAamp cador Pathogen Mini Kit (Qiagen). Quantification of the number of viral RNA copies in tissues (RNA copies/g) was similar like in other samples. IBV-positive cells in tissues were visualized and quantified using the same technique as for the cryosections of tracheal mucosa explants. Twenty consecutive cryosections of 10 μm were analyzed per tissue. The average number of infected cells was expressed per 10 mm^2^ of tissue.

#### Statistical analysis

SigmaPlot (Systat Software, Inc.) software was used to analyse the data statistically with one-way analysis of variance (ANOVA). Viral loads in tracheal secretions, plasma and mononuclear cells were log-transformed prior to analysis. The quantification limit of RT-qPCR was 3.4 log_10_ copies/mL (quantification limit is the concentration/amount of viral RNA copies, which can be quantified reliably). The detection limit of RT-qPCR was 2.4 log_10_ copies/mL (detection limit is the minimal concentration of viral that can be detected). The results of three independent experiments were shown as mean ± standard deviation (SD). *P* values of <0.05 were considered to be significant.

## Results

### IBV B1648 and M41 replication characteristics in respiratory mucosa and KUL01^+^ cells

#### B1648 and M41 replication in tracheal mucosa explants

**Figure 1:**
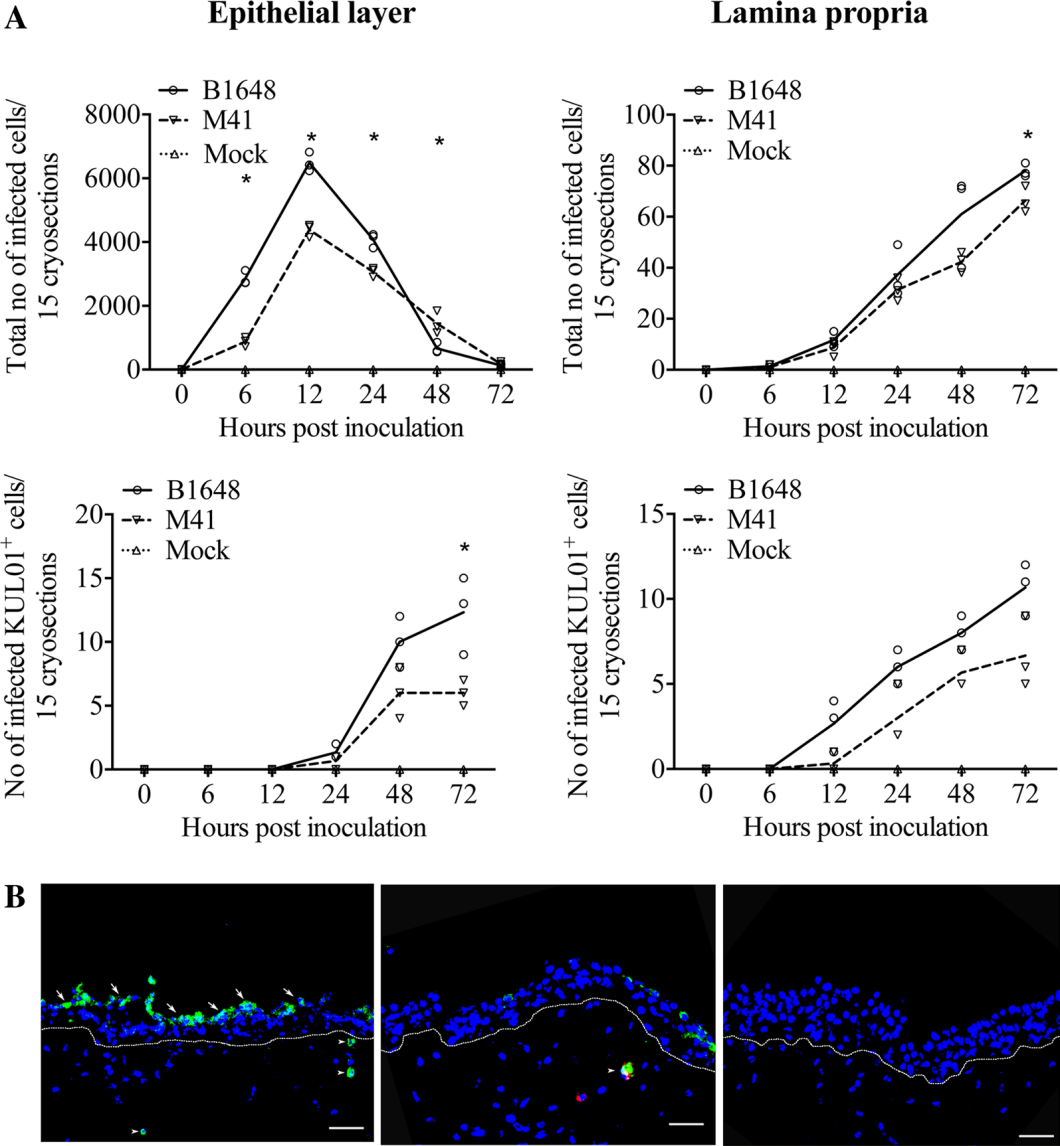
**IBV infected cell quantification (A) and antigen expression (B) in tracheal mucosa explants. A** Number of IBV B1648 and M41 infected cells and infected KUL01^+^ cells were quantified in the epithelial layer and lamina propria of tracheal mucosa explants. Fifteen consecutive cryosections were analysed at 0, 6, 12, 24, 48 and 72 hpi to quantify infected cells. The number of infected KUL01^+^cells starts to increase from 12 hpi, when the total number of IBV infected cells starts to decrease. Lines represent the evolution of arithmetic means without any transformation of original data. Solid line represents mean values of B1648, dashed line represents mean values of M41 and dotted line represents mean values of mock. An asterisk (*) indicates a significant difference (*P* < 0.05) between B1648 and M41. **B** Representative confocal photomicrograph illustrating IBV infected cells (left panel) in the epithelium (arrows) and lamina propria (arrowheads), and IBV infected KUL01^+^cell (middle panel) in lamina propria (arrowhead) of the tracheal mucosa. Mock-inoculated tracheal mucosa (right panel). Green fluorescence visualises IBV antigens. KUL01^+^ cells are visualised by red fluorescence. Cell nuclei were stained with Hoechst (blue). White dotted line indicates the BM. Scale bar represents 50 μm.

Inoculation of chicken tracheal mucosa explants with IBV B1648 and M41 led to the appearance of viral antigen positive (infected) cells in the epithelium and lamina propria of the tracheal mucosa starting from 6 hpi (Figure 1). In the epithelial layer of the tracheal mucosa, the number of B1648 and M41 infected cells gradually increased until 12 hpi, and later gradually decreased up to 72 hpi (Figure 1A). The number of B1648 infected cells in the epithelium was significantly higher than M41 infected cells at 6 [B1648: 2856.3 ± 217.2 (which equals 37.2 ± 2.8% of the epithelial cells), M41: 877.0 ± 142.7 (11.2 ± 2.9%), *P* = 0.0002)], 12 [B1648: 6490.3 ± 298.9 (82.5 ± 4.1%), M41: 4380.0 ± 203.1 (57.6 ± 3.4%), *P* = 0.0005] and 24 hpi [B1648: 4077.3 ± 225.4 (51.8 ± 4.8%), M41: 3064.3 ± 144.9 (38.9 ± 3.0%), *P* = 0.0028]. The number of M41 infected cells was significantly higher than B1648 infected cells at 48 hpi (B1648: 664.7 ± 164.7, M41: 1446.7 ± 354.3, *P* = 0.0257) and were slightly higher at 72 hpi (B1648: 130.0 ± 62.4, M41: 179.3 ± 67.6, *P* = 0.4057). From 48 hpi, the epithelial layer was almost totally gone, which explained the drop of positive cells.

In the lamina propria of the tracheal mucosa, B1648 and M41 infected cells were visible from 6 hpi and increased in number over time (Figure 1A). In the lamina propria, the average number of B1648 infected cells was only slightly higher than M41 infected cells at 6, 12, 24 and 48 hpi (*P* = 0.6433–0.1578) but significantly higher at 72 hpi (*P* = 0.0249).

Double immunofluorescence staining was performed for the identification of IBV susceptible cells. IBV infected KUL01^+^ cells were detected in the epithelium and lamina propria of the respiratory mucosa inoculated with both B1648 and M41 (Figure 1A). In the epithelium, the average number of B1648 infected KUL01^+^ cells was slightly higher than M41 infected KUL01^+^ cells at 24 and 48 hpi (*P* = 0.2302–0.0705) but significantly higher at 72 hpi (*P* = 0.0270). In the lamina propria, the average number of B1648 infected KUL01^+^ cells was higher than M41 infected KUL01^+^ cells at 12, 24, 48 and 72 hpi (*P* = 0.0686–0.0550). Interestingly, the number of infected KUL01^+^ cells (monocytic cells) started to increase from 12 hpi, when the total number of IBV infected cells started to decrease. Representative images of IBV infected cells and IBV infected KUL01^+^ cells are given in Figure 1B.

The above results indicate that in the epithelium B1648 replicated better than M41 until 24 hpi. Then, the replication pattern changed. B1648 exploited more infected KUL01^+^ cells but without significant differences with M41 in most of the times post inoculation evaluated.

#### IBV B1648 and M41 replication in blood monocytic cells (KUL01^+^ cells)

**Figure 2:**
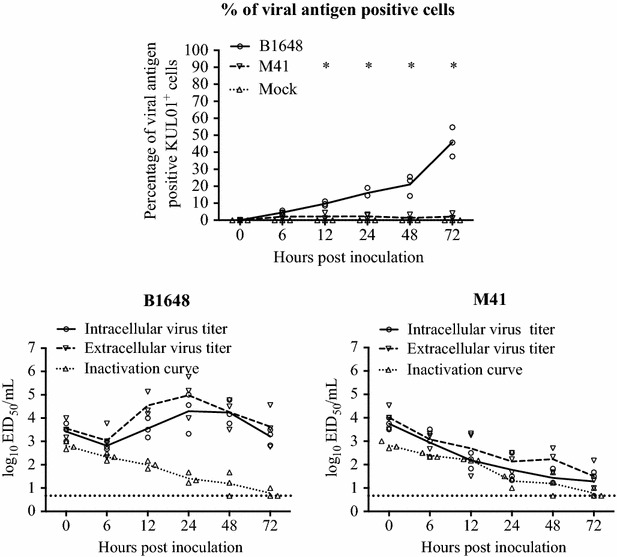
**IBV replication kinetics in blood monocytes (KUL01** ^**+**^ **cells).** The monocytes were inoculated with IBV B1648 or M41 at a m.o.i. = 5. Percentage of viral antigen positive KUL01^+^ cells was determined in B1648 and M41 infected blood mononuclear cells at 0, 6, 12, 24, 48 and 72 hpi by double immunofluorescence. For viral antigen positive cells, the solid line represents the mean values of the B1648 group, the dashed line represents the mean values of the M41 group and the dotted line represents the mean values of the mock group. Intracellular and extracellular virus titers of cell lysate and supernatant were determined at designated time points. For viral titers, the solid line represents the mean values of the intracellular virus titers, the dashed line represents the mean values of the extracellular virus titers and the dotted line represents the mean values of the inactivation curve. An asterisk (*) indicates a significant difference of viral antigen positive KUL01^+^ cells between B1648 and M41 (*P* < 0.05). The inactivation curve shows the drop of virus titers at 37 °C in culture medium due to inactivation events.

The replication kinetics of IBV B1648 and M41 were compared in the peripheral blood monocytic cells (KUL01^+^ cells) (Figure 2). In blood monocytes (KUL01^+^ cells), the percentage of B1648 infected cells was significantly higher than M41 infected cells at 12 (B1648: 9.7 ± 1.3%, M41: 2.2 ± 1.9%, *P* = 0.0051), 24 (B1648: 16.0 ± 2.6%, M41: 2.3 ± 1.5%, *P* = 0.0013), 48 (B1648: 21.1 ± 6.0%, M41: 1.4 ± 1.7%, *P* = 0.0053) and 72 hpi (B1648: 46.0 ± 8.6%, M41: 2.2 ± 1.7%, *P* = 0.0010). Virus titers were analyzed in the cell lysate (intracellular virus) and supernatant (extracellular virus) of the infected blood monocytes. The intracellular and extracellular B1648 virus titers increased over time until 24 hpi, and later reached a plateau or slightly decreased up to 72 hpi. The intracellular virus titers with strain B1648 were significantly higher than M41 strain at 12 (*P* = 0.0115), 24 (*P* = 0.0152), 48 (*P* = 0.0014) and 72 hpi (*P* = 0.0063). The extracellular titers with B1648 were higher than with M41 at 12 hpi (*P* = 0.0501), and were significantly higher at 24 (*P* = 0.0077), 48 (*P* = 0.0118) and 72 hpi (*P* = 0.0270). On the contrary, no increase in viral titers was seen in cell lysate and supernatant of blood monocytic cells infected with M41, with a slope comparable to the inactivation curve. Over all, the above results demonstrated that only B1648 could establish a productive infection in the peripheral blood monocytic cells (KUL01^+^ cells).

### Replication kinetics between B1648 and M41 strains in chickens

#### Clinical signs and water consumption

After inoculation with B1648 and M41, all chickens showed signs of illness characterized by depression, ruffled feathers, huddling together, tracheal rales, sneezing, coughing and dyspnoea from 2 to 10 dpi. None of the control chickens showed any clinical signs during the whole experiment. Water consumption was increased in the B1648 group (1370.7 g/bird) compared to the control group (853.7 g/bird) and the M41 group (792.2 g/bird). Reduced body weight gain was observed in the B1648 group compared to the control group and the M41 group (data not shown).

#### Post mortem findings

**Figure 3:**
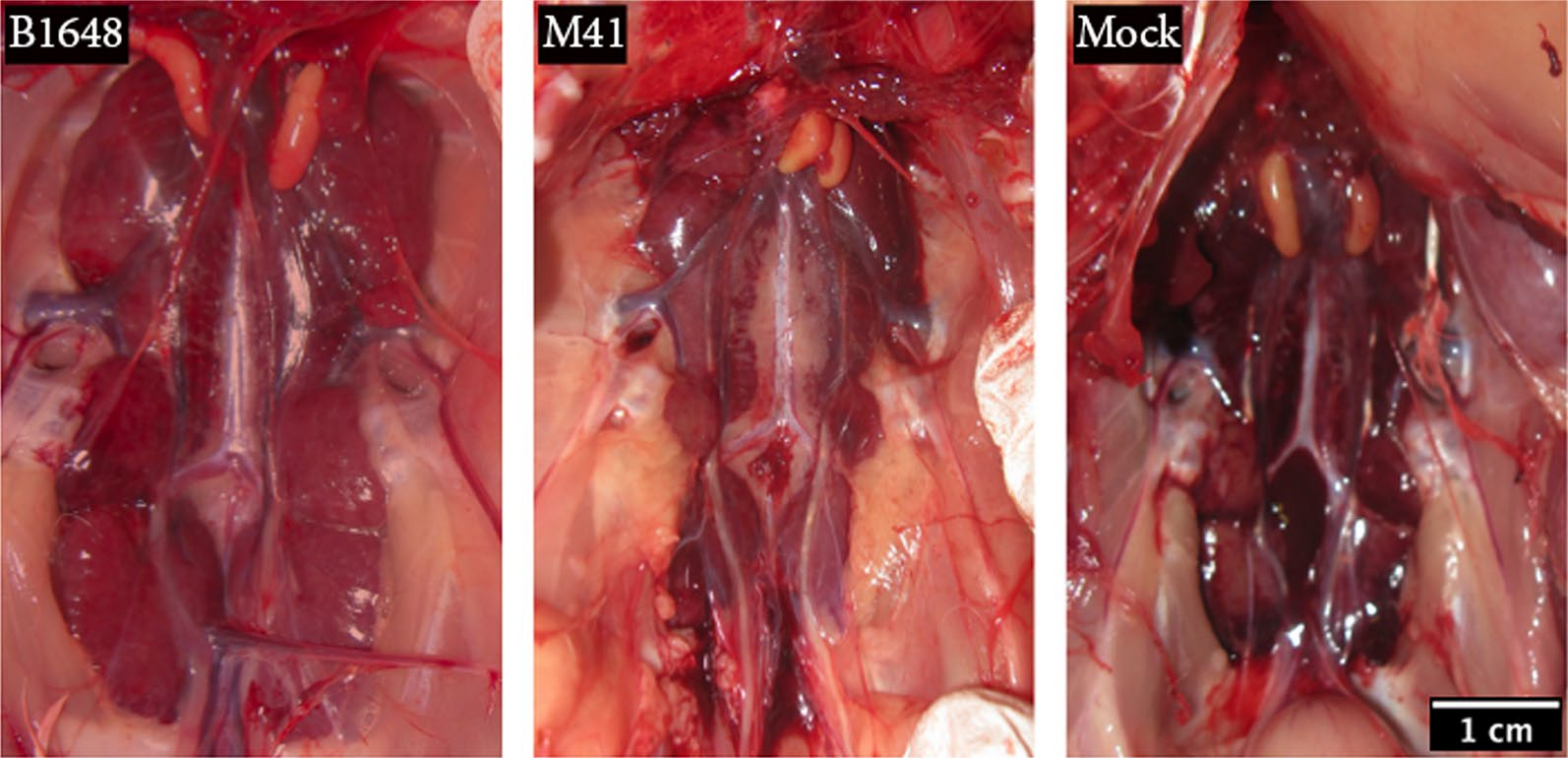
**Representative photographs of kidneys after euthanasia (12** **dpi).** Kidneys were collected from chickens inoculated with IBV B1648 and M41, and PBS (mock). The kidneys of the chicken infected with B1648 were clearly enlarged.

Chickens were euthanized at 12 dpi. Tissue samples of trachea, lungs, liver, spleen and kidneys were collected from all three groups. At necropsy, enlarged kidneys were observed in animals of the B1648 group. No lesions were observed in kidneys of the M41 group (Figure 3). Gross lesions were not observed in trachea, lungs, liver and spleen of animals of the B1648, M41 and control groups.

#### Standardization of SYBR green-based RT-qPCR assay for ORF 1a gene and interpretation of results

**Figure 4:**
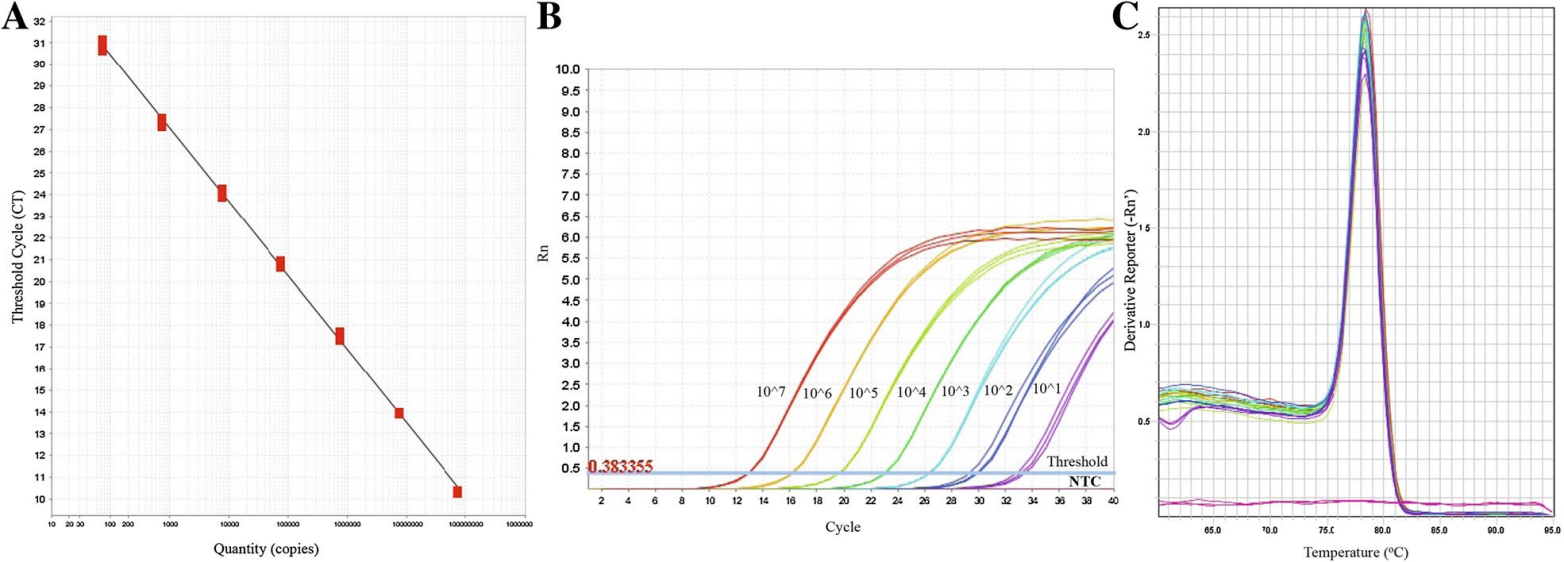
**Standardization of SYBR green-based RT-qPCR for** ***ORF 1a gene.*** **A** Standard curve, **B** amplification plot and **C** melt curve analysis over a linear dynamic range from 1 log_10_ to 7 log_10_ copies/reaction.

SYBR green-based one step RT-qPCR was performed to generate a standard curve, using 1:10 serially diluted standard RNA templates. The results indicated that the standard curve had a wide dynamic range (10^1^–10^7^ copies/reaction) with the high linear correlation (R^2^ = 0.9999) between the cycle threshold (Ct) value and template concentration (Figure 4A). The slope of the standard curve was −3.297. The amplification efficiency of the RT-qPCR assay was 102.3%, according to the slope of the exponential phase in the amplification chart (Figure 4B). Melt curve analysis showed amplification of a specific product with a melting peak at 78.31 °C (Figure 4C), which was confirmed by the agarose gel electrophoresis analysis. Amplification was not observed in the non-template control. RNA extracts from samples were analyzed in duplicate reactions. Quantification of the viral RNA copies was possible if the Cq values of both reactions fell within the LDR of the RT-qPCR assay. Samples with a specific melt curve were considered positive but not quantifiable when the Cq value of the reactions fell at Cq values higher than the lowest peak of the LDR.

#### RT-qPCR for tracheal secretions, plasma and mononuclear cells

**Figure 5:**
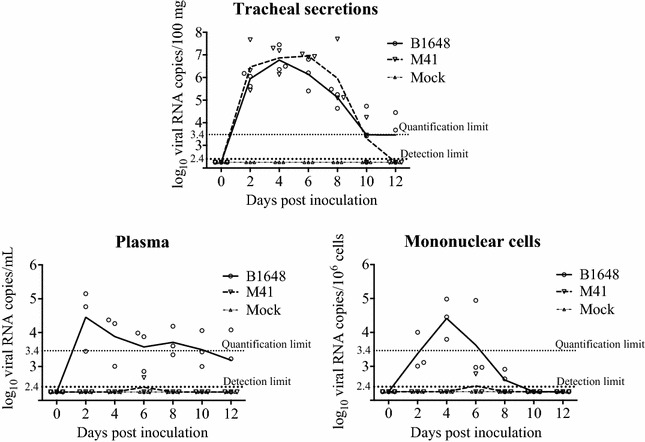
**IBV RNA in tracheal secretions, plasma and mononuclear cells.** Samples were collected from IBV B1648 and M41, and PBS (mock) inoculated chickens at 0, 2, 4, 6, 8, 10 and 12 dpi. Viral RNA was measured by RT-qPCR. Solid and dashed lines represent mean viral RNA copies per strain and per day. Mock is represented by the dash-dotted line. The quantification limit of RT-qPCR was 3.4 log_10_ copies/mL (dotted line). The detection limit of RT-qPCR was 2.4 log_10_ copies/mL (bold dotted line).

Viral RNA in tracheal secretions, plasma and mononuclear cells was quantified by RT-qPCR (Figure 5). The virus-shedding pattern in tracheal mucosa was the same until 10 dpi for both B1648 and M41. Viral shedding in tracheal mucosa was identified from 2 to 12 dpi in the B1648 group with a maximum number of 10^6.8^ viral RNA copies/100 mg, and from 2 to 10 dpi in the M41 group with a maximum number of 10^7.0^ viral RNA copies/100 mg. In the B1648 group, two chickens were positive at 10 and 12 dpi. Two chickens were positive at 1 dpi, in the M41 group. In tracheal secretions, viral RNA copies were not significantly different between the B1648 and M41 groups.

In plasma, viral RNA was first detected in the B1648 group at 2 dpi and was observed until the end of the study (12 dpi, *n* = 2), with a peak number of 10^4.5^ viral RNA copies/mL. In the M41 group, viral RNA was not detected in plasma, except of one animal at  dpi (*n* = 1, 10^2.6^ viral RNA copies/mL).

In mononuclear cells, viral RNA was first detected in the B1648 group at  dpi and lasted until 8 dpi (*n* = 2) with a peak number of 10^4.4^ viral RNA copies/10^6^ cells. In contrast, viral RNA was not detected in mononuclear cells of the M41 inoculated animals, except of one chicken at 6 dpi (*n* = 1, 10^2.8^ viral RNA copies/10^6^ cells).

The statistical analysis was not performed on viral RNA copies in plasma and mononuclear cells because the M41 group was negative except of one chicken at 6 dpi.

#### Immunostainings of cytospinned mononuclear cells

**Figure 6:**
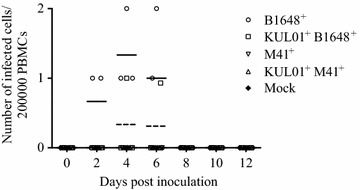
**Quantification and identification of IBV B1648 and M41 infected total and KUL01** ^**+**^ **PBMC.** IBV B1648 and M41, and PBS (mock) inoculated blood samples were collected at 0, 2, 4, 6, 8, 10 and 12 dpi; 200 000 mononuclear cells were cytospinned and immunostainings were performed. B1648^+^ infected PBMC were observed at 2, 4 and 6 dpi and KUL01^+^B1648^+^ infected cells were observed at 4 and 6 dpi.

Immunofluorescence stainings of 200 000 cytospinned mononuclear cells revealed B1648 infected cells at 2 (2/3 animals), 4 (3/3) and 6 (2/3) dpi, and B1648 infected KUL01^+^ cells at 4 (1/3) and 6 (1/3) dpi. Infected cells were not observed in cytospinned mononuclear cells of M41 and control groups (Figure 6).

#### Virus isolation in plasma and mononuclear cells

To demonstrate the presence of infectious virus from plasma and mononuclear cells of infected animals, virus isolation was performed by several passages on 10 days old embryonated B1648 inoculated eggs [22]. After two blind passages, plasma of all B1648 inoculated animals at 2, 4, 6, 8, 10 and 12 dpi and mononuclear cells of all B1648 inoculated animals at 2, 4, 6 and 8 dpi caused clear dwarfing and curling of inoculated embryos (Table 3). With mononuclear cells of the B1648 group, lesions were observed in one out of three chickens at 10 and 12 dpi, after two blind passages. Even after five blind passages, plasma and mononuclear cells from the M41 group showed no embryonic lesions, demonstrating the absence of infectious virus.

**Table 3:** **Virus isolation of plasma and mononuclear cells**

Samples	IBV strain	Number of positive animals per three inoculated animals at … days post inoculation						
		0	2	4	6	8	10	12
Plasma	B1648	0/3	3/3	3/3	3/3	3/3	3/3	3/3
	M41	0/3	0/3	0/3	0/3	0/3	0/3	0/3
	Control	0/3	0/3	0/3	0/3	0/3	0/3	0/3
Mononuclear cells	B1648	0/3	3/3	3/3	3/3	3/3	1/3	1/3
	M41	0/3	0/3	0/3	0/3	0/3	0/3	0/3
	Control	0/3	0/3	0/3	0/3	0/3	0/3	0/3

Plasma and mononuclear cells were collected from chickens at different time points post inoculation with IBV B1648 and M41. Virus isolation was performed by inoculating embryonated eggs.

#### RT-qPCR and immunofluorescence staining of tissues

**Figure 7:**
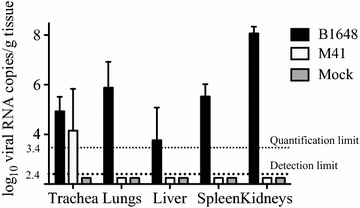
**Viral RNA copies in the trachea, lungs, liver, spleen and kidneys at 12 dpi.** Tissues were collected at euthanasia (12 dpi) from chickens inoculated with IBV B1648 and M41, and PBS (mock). Viral RNA copies (log_10_ viral RNA copies/g of tissue) were measured by RT-qPCR. The quantification limit of RT-qPCR was 3.4 log_10_ copies/mL (dotted line). The detection limit of RT-qPCR was 2.4 log_10_ copies/mL (bold dotted line).

**Figure 8:**
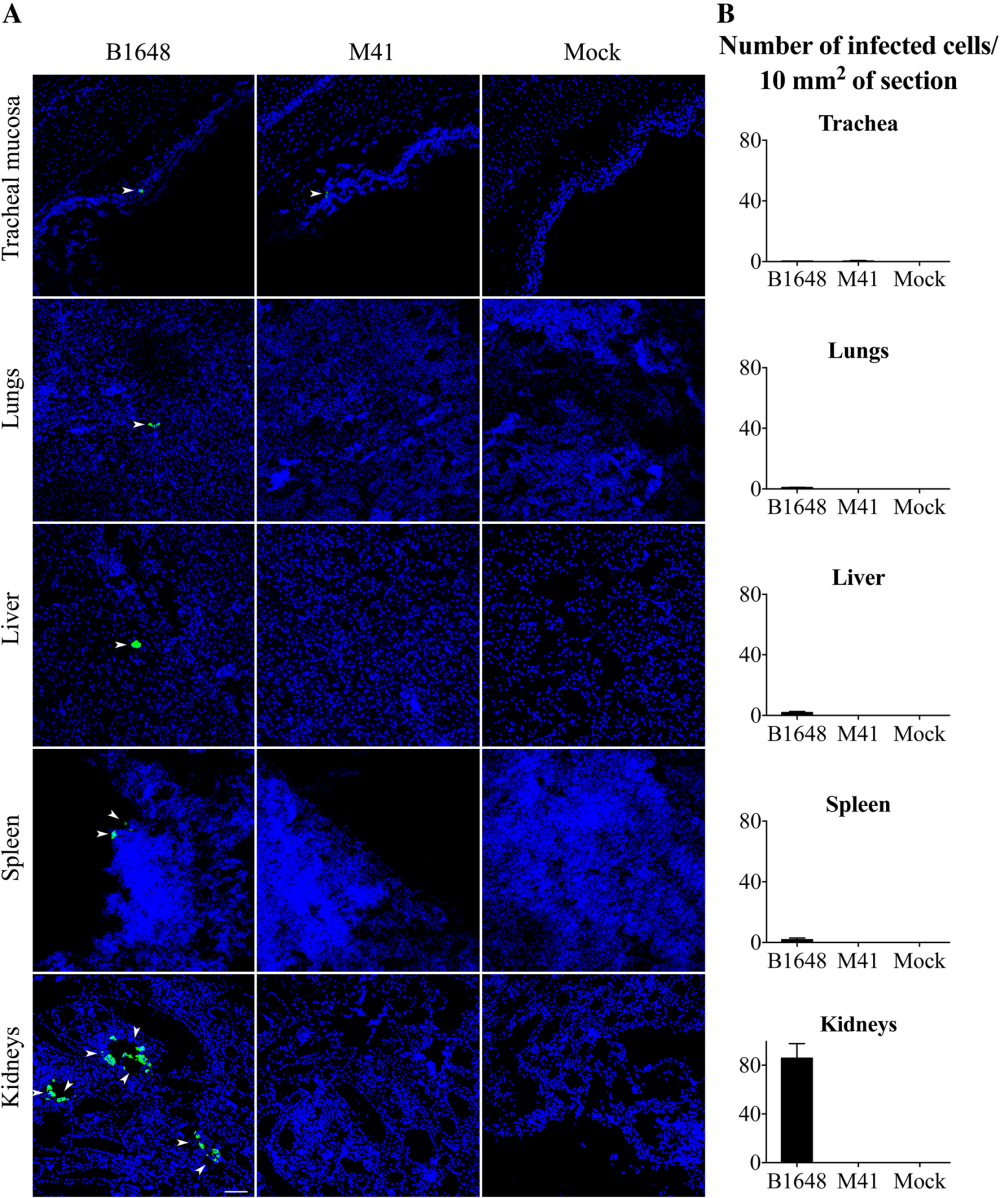
**IBV infected cell localization (A) and quantification (B) in various tissues at 12 dpi.** Trachea, lungs, liver, spleen and kidneys were collected from IBV B1648 and M41, and PBS (mock) inoculated chickens at 12 dpi. **A** Representative confocal photomicrographs illustrating viral antigen positive cells (arrowheads). Green fluorescence visualises IBV antigens. Cell nuclei were stained with Hoechst (blue). Scale bar represents 50 μm. **B** Quantification of viral antigen positive cells was performed per 20 consecutive cryosections of 10 μm tissue. Viral antigen positive cells were quantified in the tracheal mucosa of B1648 and M41 infected animals and in the lungs, liver, spleen and kidneys of only B1648 infected animals. The average number of viral antigen positive cells present in different tissues is presented per 10 μm^2^. For each tissue, mean and standard deviation (SD) are shown.

Viral RNA was measured in samples of trachea, lungs, liver, spleen, and kidneys by RT-qPCR (Figure 7). Viral RNA was present in all collected tissues of the B1648 group and, only in tracheal samples of the M41 group (B1648: 10^4.9 ± 0.5^ copies/g, M41: 10^3.4 ± 0.5^ copies/g). The average B1648 viral RNA content in the lungs, liver, spleen and kidneys were 10^5.8 ± 1.0^, 10^3.0 ± 2.6^, 10^5.5 ± 0.5^ and 10^8.1 ± 0.3^ copies/g, respectively. Immunofluorescence staining was performed on tissues to quantify viral antigen positive cells (Figure 8). IBV infected cells were detected in the tracheal mucosa of animals inoculated with both strains (B1648: 0.33 ± 0.06/10 mm^2^, M41: 0.40 ± 0.33/10 mm^2^) and in the lungs (0.96 ± 0.13/10 mm^2^), liver (1.86 ± 0.80/10 mm^2^), spleen (1.81 ± 1.16/10 mm^2^) and kidneys (85.71 ± 12.06/10 mm^2^) of only animals inoculated with B1648.

## Discussion

Despite the circulation of nephropathogenic and respiratory IBV strains for over five to six decades, it is not well understood why some IBV strains have a kidney tropism [3, 7, 15]. Hence, we have investigated the replication patterns of IBV nephropathogenic (B1648) and respiratory (M41) strains in vitro, in respiratory mucosa explants and peripheral blood monocytes, and in chickens.

The replication kinetics and invasion strategies of IBV B1648 and M41 were better understood by the work done with respiratory mucosa explants. Similar to many other IBV viruses, B1648 and M41 are epitheliotropic in the respiratory mucosa [3, 5, 7]. Replication kinetics of B1648 and M41 in the respiratory mucosa explants have revealed some important differences between these strains. B1648 replicated somewhat better than M41 in the epithelium of the respiratory mucosa at 6, 12 and 24 hpi (*P* < 0.05). Consequently, B1648 destroyed the epithelial layer more severely than M41. The desquamation of the cells present in the epithelial layer is the main reason for the decreased number of IBV infected cells after 24 hpi [30]. Above results indicated that B1648 was highly virulent compared to M41 in the epithelial layer of respiratory mucosa.

There have been several reports on the virulence of IBV strains in in vitro tracheal organ cultures. Chhabra et al. [31] and Raj and Jones [32] have reported that in tracheal organ cultures, the M41 strain was highly pathogenic compared to the variant nephropathogenic IS/885/00-like and QX-like IBV strains and other variant strains (Strain G and strain 6/1432/81) [9, 33]. In addition, Abd El Rahman et al. [34] have shown that Beaudette (highly passaged Massachusetts-derived strain) and QX were highly pathogenic compared to Italy 02 and 4/91 variant strains. According to the above in vitro reports, M41 strain used in the present study seems to be somewhat less virulent than other M41 strains reported in literature [31, 32].

Although speculative, a hypothesis can be formed to explain the lower virulence of M41 strain in vitro, which was used in the present study. The passage history of the strain and genome sequence should be considered. The passage history of the present study M41 strain is unknown [21, 22]. For IBV isolation from infected chickens, sometimes several passages of the strain in embryonated eggs are necessary to observe characteristic clinical signs of infection on embryos [22]. The repeated passages may alter the virulence and antigenicity of the virus [22]. If the present study M41 strain has been serially passaged in the past, then it may have lost part of its replication ability, and it may not be able to replicate or show virulence to the extent that is associated with other reported M41 strains [31, 32]. Therefore, it is important to give more emphasis on the passage history of the strains to have a better understanding about their virulence and its associated biological properties [35]. The genome sequence of the present study M41 strain may be different from those of the M41 strains reported by Chhabra et al. [31], Raj and Jones [32] and Abd El Rahman et al. [34]. At present, there is no information available on the full genome sequence of M41 strain used in the present study. In the future, our M41 strain will be sequenced and analyzed. However, in contrast with the in vitro findings, our M41 was replicating better than the B1648 IBV strain in vivo demonstrating the fitness of our M41 in birds. Therefore, one should be careful with overinterpreting the in vitro results.

Similar replication differences were observed in tracheal organ cultures with many other IBV strains and serotypes [32, 36, 37]. In general, the differences in virulence of strains at the respiratory mucosa might be related to the presence/absence, and/or differential expression of receptors on susceptible epithelial cells, antiviral responses of the host cells and immune-evasion mechanisms developed by the IBV strain [38]. It would be interesting to compare the replication kinetics in in vitro tracheal mucosa explants, between B1648 and other worldwide important IBV strains such as variant nephropathogenic IS/885/00-like and QX-like strains, recent variant M41 strains and variant Italy 02 and 4/91 strains [7].

During the epithelial cell layer infection, both B1648 and M41 infected cells penetrated through the basement membrane (BM) into the lamina propria. The number of B1648 viral antigen positive cells in the lamina propria was quite higher than the number of M41 viral antigen positive cells at 6, 12, 24 and 48 hpi, and was significantly higher at 72 hpi (*P* < 0.05). Certain infected cells were identified as KUL01^+^ cells (monocytic cells) and, whose number started to increase from 12 hpi, where the total number of IBV infected cells started to decrease. It could be that the infected monocytic cells (KUL01^+^ cells) that increased after 12 hpi, started to cross the BM to enter the lamina propria and underlying layers. These infected monocytic cells (KUL01^+^ cells) may play a crucial role in dissemination of virus to the blood circulation and internal organs. Therefore, monocytic cells may be identified as carrier cells that breach through the BM to reach underlying layers of the respiratory mucosa. The use of monocytic cells by nephropathogenic IBV as a Trojan horse to penetrate through connective tissues is in line with what has been observed with equine herpes virus 1 (EHV1) and European equine arteritis virus in the respiratory mucosa of horses [16, 17, 39, 40]. In our study, many infected cells in the epithelium and lamina propria were observed. Because only a certain percentage was identified as KUL01^+^, efforts will be made to identify the KUL01 negative IBV infected cells in the propria of tracheal mucosa explants.

The percentage of infected peripheral blood monocytes (KUL01^+^ cells) and virus production was higher (*P* < 0.05) with B1648 than with M41 at 12, 24, 48 and 72 hpi. The intracellular and extracellular titers showed that B1648 has a strong productive replication in the peripheral blood monocytes. The decrease or no change in the intracellular and extracellular virus titers of B1648 strain at 48 and 72 hpi may be due to the absence of available susceptible cells or due to an antiviral response (e.g. interferon). This is in line with the MERS-CoV and dengue virus interactions with human macrophages, and classical swine fever virus interactions with monocyte derived dendritic cells [41–43]. Absence of increase of percentage of infected cells and virus titers in the cell lysate and supernatant with M41 demonstrated that the M41 infection of monocytes was abortive. The productive replication of B1648 and abortive replication of M41 is most probably the result of differences at certain steps of the replication cycle (entry, disassembly, genome release, transcription, translation and assembly). This issue will be further explored in the future.

In general, coughing, dyspnea, tracheal rales, depression, ruffled feathers and huddling together are characteristic clinical signs of IB in chickens [1]. Increased water consumption is a typical clinical sign of nephropathogenic IB and was recorded in the B1648 group [9, 30]. Reduced body weight gain was another important clinical sign noticed in the B1648 group (data not shown). At postmortem, swollen kidneys were principal macroscopic lesions recorded in the B1648 group, which is consistent with other nephropathogenic strains [1].

RT-qPCR is a reproducible and reliable method for rapid quantification of IBV RNA in clinical samples [44–46]. At present, most of the available RT-qPCR assays, which target the nucleocapsid gene, 5′ untranslated region, or the *S1* gene of the IBV genome are not efficient to quantify B1648 and M41 strains in a consistent way, by the highly flexible and cost effective SYBR green method [44–46]. Other RT-qPCR assays that target the 3′ end of the viral genome [47–49], usually give a 3–4 log_10_ overestimation of viral genomic RNA, as this 3′ RT-qPCR detects not only genomic RNA, but also all subgenomic mRNAs [19]. Indeed, during coronavirus replication, RNA dependent RNA polymerase synthesizes genomic RNA, as well as minus strand and subgenomic mRNAs. Based on our past experience with feline coronavirus quantification, 5′ RT-qPCR is the most consistent and reliable quantification method, because it evades overestimation of genomic RNAs than 3′RT-qPCR [19, 50]. Thus, 5′ RT-qPCR was developed to avoid quantification of the subgenomic mRNAs.

After inoculation of virus, both B1648 and M41 initiate extensive virus replication in the epithelium of the upper respiratory tract (URT) and shed virus in the respiratory secretions between 2 to 12 dpi. In contrast to the in vitro results in tracheal mucosa explants, the replication of B1648 and M41 were very similar except that B1648 replication was persisting longer (12 dpi). It would have been interesting, if tracheal swabs and mucosal tissues were collected at 6, 12, 24, 48 and 72 hpi (similar to in vitro experiments) to better understand the infection kinetics at the epithelial layer and lamina propria layer of in vivo tracheal mucosa and to compare it with in vitro results. The primary local replication of both B1648 and M41 at the URT allows horizontal transmission virus to susceptible birds and ensures virus circulation in birds [51].

Viral RNA was detected in plasma of B1648 inoculated animals at 2 (*n* = 3), 4 (*n* = 3), 6 (*n* = 3), 8 (*n* = 3), 10 (*n* = 3) and 12 dpi (*n* = 2) at a concentration of 10^2.4–4.5^ and in mononuclear cells of B1648 inoculated animals at 2 (*n* = 3), 4 (*n* = 3), 6 (*n* = 3) and 8 (*n* = 2) dpi at a concentration of 10^1.8–4.4^ viral RNA copies/10^6^ mononuclear cells. These B1648 viral RNA copies of plasma and mononuclear cells were originated from infectious virus as confirmed by virus isolation. In M41 inoculated animals, viral RNA was present in only one animal at 6 dpi in plasma and mononuclear cells but no infectious virus could be demonstrated by virus isolation. Infected cells were demonstrated by immunofluorescence in cytospinned mononuclear cells at 2 (*n* = 2), 4 (*n* = 3) and 6 dpi (*n* = 2) with B1648. M41 infected cells were never demonstrated. These results demonstrated the onset of a cell free and cell-associated viremia at 2 dpi with B1648. This indicates that cell free B1648 virus and/or B1648 infected cells are able to enter into the blood circulation very quickly. The potential spread of B1648 from blood into the internal organs was demonstrated. In contrast with B1648, infectious virus was not demonstrated for M41 to reach the blood circulation and internal organs. Pseudorabies virus and classical swine fever virus are other examples of viruses that result in a combination of cell free and cell-associated viremia leading to the viral spread in mammals [52, 53]. For many avian viral infections, there is no information on the viremic phase and its pathogenic consequences.

The infected mononuclear cells were mainly KUL01^+^ cells but also some KUL01 negative cells were present. The latter were not identified. They could be unidentified blood monocytic cells (KUL01 negative), B and T lymphocytes. The KUL01 marker is not reported to identify all the monocytic cells of the blood circulation [24]. Hence, the role of blood dendritic cells, KUL01 negative monocytic cells, B and T lymphocytes in B1648 infection kinetics and dissemination needs to be elucidated in future work.

In the B1648 group, cell-free virus in plasma persisted until the end of the study (12 dpi). Different sources may have continuously contributed to the cell-free virus in plasma during the course of B1648 infection. At early time points (2 and 4 dpi), cell free virions produced by tracheal epithelial cells may have infiltrated into the underlying submucosal layers. Then, through capillaries cell-free virions may have reached blood circulation. At 6 and 8 dpi, infected blood mononuclear cells may have released virus in plasma. The presence of infectious virus in plasma at later time points (10 and 12 dpi) but not in mononuclear cells indicated that cell free virions from infected internal organs may have leaked into the blood circulation. In the M41 group, the absence of viral RNA and infectious virus, especially at later time points (8, 10 and 12 dpi), is also an indication that infectious virus could not have reached internal organs.

Viral RNA and antigen positive cells were only detected in lungs, liver, spleen and kidneys of B1648 infected animals at 12 dpi, demonstrating that only B1648 was disseminated through the mononuclear cells and plasma but not M41 at that stage of infection. In the M41 group, the possibility of presence of viral RNA and antigen positive cells in tissues at earlier time points (6, 8 and 10 dpi) cannot be completely excluded. B1648 replicated more productively in the kidneys compared to lungs, liver and spleen, illustrating the high nephropathogenicity of B1648 [7, 13, 14]. Infected KUL01^+^ cells (colocalization) were not observed in the tissues. This demonstrates that once the virus reached internal organs, it is replicating in non-KUL01^+^ cells [7]. In kidneys it is known that it replicates in tubular cells.

In this study, we observed several differences between B1648 and M41 strains at the level of mononuclear cells (KUL01^+^ cells). Firstly, the number of B1648 infected KUL01^+^ cells in the lamina propria of in vitro tracheal mucosa explants was quite higher than M41. Secondly, the percentage of infected cells and virus production in the in vitro inoculated blood mononuclear cells (KUL01^+^ cells) was higher for B1648 than for M41. Thirdly, viral RNA copies were detected in mononuclear cells of all B1648 infected chickens and exceptionally with M41. The results above implicate that monocytic cells may be important carrier cells, which are required for the progress of B1648 disease. The productive and sustainable replication of B1648 virus in blood monocytic cells (KUL01^+^ cells) may represent an important strategy used by B1648 to disseminate virus to the kidneys and other internal organs. In mammals, many highly pathogenic viruses or virus strains exploit and replicate productively in macrophages to cause severe systemic disease [43, 54–56]. Because monocytes/macrophages are usually the first line of defense against virus entry, the successful replication of virus in these cells may hamper their immunological functions and by the migratory characteristics of these cells, virus is disseminated throughout the body. In this context, monocytes are considered as double edged swords [57–59].

Even though M41 infected KUL01^+^ cells were observed in the lamina propria of the tracheal mucosa explants, the abortive replication of M41 in monocytic cells (KUL01^+^ cells) most probably hampered the dissemination of M41 via blood to reach the kidneys and other internal organs. Furthermore, the replication ability of M41 may be lowest in the lamina propria and underlying layers to release cell free virus. Therefore, it can be stated that the M41 strain that was used in the present study can replicate only in the respiratory mucosa, and cannot spread to the internal organs for further disease progression. The results presented here are somewhat different with those from other reports, where Massachusetts type strains (M41) were detected in kidneys [60, 61]. Some other studies with the M41 reference strain have shown only mild histopathological lesions with the absence of viral antigen positive cells [62]. The basis for the differences between the different studies is not clear but might be due to different M41 strains, passage levels, experimental design and conditions, nutrition, environment, intercurrent infections, age/breed of the birds, and detection techniques [30, 63].

Otherwise, M41 strains used in recent reports might be mutants/variants of the prototype M41. Mutation and recombination events are common in coronaviruses, which are likely to contribute to the emergence of new variants/mutants and outbreaks of new diseases [64]. Currently, at least 8 full genome sequences of M41 are available in Genbank. The full genome sequence of the latest M41_2006 was closest (90.8%) to the B1648 full genome sequence compared to earlier Mass 41_1965-85 (89.7%) full genome sequences [15]. It could be that the recent M41 strains may be recombinant viruses like M41_2006 with only the spike gene from the prototype M41 and the remaining genome regions from Connecticut and California serotypes [65]. The Mass type (M41) is identified/classified based on the sequence of its spike gene (3500 bp) only, but one should consider that remaining genomic regions (24 000 bp) may also play an important role in the pathogenicity of the virus [15, 66]. Thus, in the near future, full sequences should be considered and not only parts of it. Therefore, the full genome sequence of the M41 strain used in the present study will be elucidated to understand its evolutionary relationship with other Massachusetts type strains.

The cell-associated viremia in mononuclear leukocytes and cell free virus in plasma and the replication in kidneys of B1648 virus infected chickens is very similar to another coronavirus, MERS-CoV in humans. MERS-CoV was also reported in whole blood and plasma of humans [67–69]. In addition, MERS-CoV is replicating in monocytic cells and kidneys, resulting in high mortality in humans [43, 67–70].

**Figure 9:**
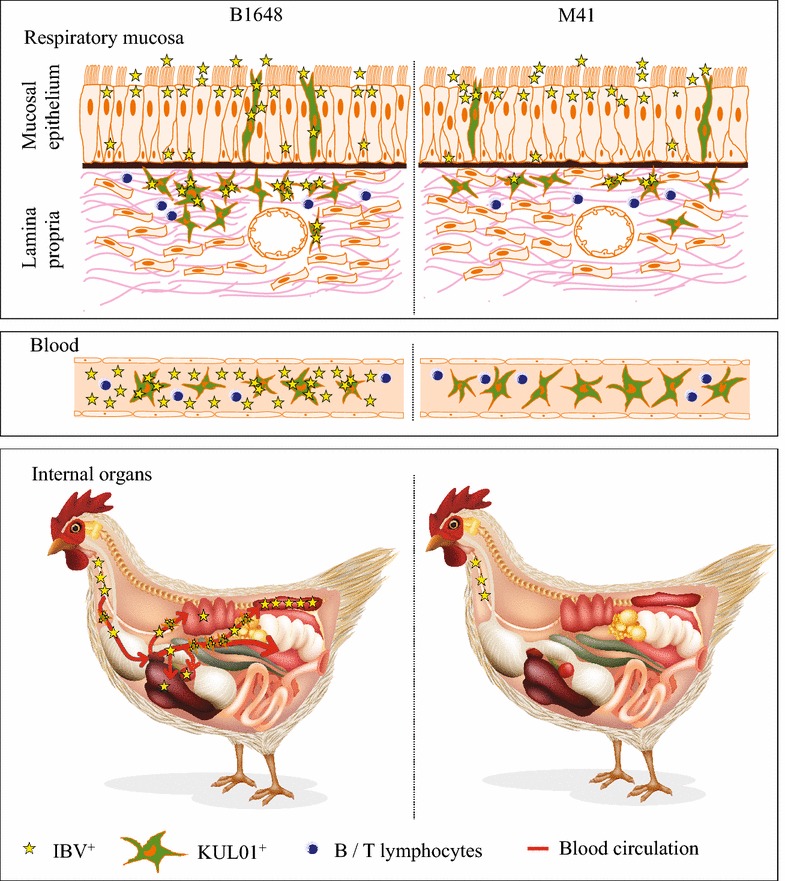
**Hypothetical model of IBV B1648 and M41 replication kinetics in respiratory mucosa, blood and internal organs of chickens.** Stars represent infectious virus. Star number and infectivity are directly proportional to each other. After natural entry of virus through nostrils, both nephropathogenic IBV B1648 and M41 replicate in the tracheal mucosa. B1648 uses a higher number of infected KUL01^+^ cells as carriers to penetrate deeper in the layers of the respiratory mucosa in contrast to M41. B1648 productively infects KUL01^+^ blood monocytic cells while M41 infection is abortive. Only B1648 disseminates via a cell-associated viremia in mononuclear leukocytes and cell free virus in plasma to reach lungs, liver, spleen and kidneys. B1648 productive infection is present in kidneys and to a less degree in lungs, liver and spleen.

In summary, B1648 and M41 strains followed different tissue tropism mechanisms (Figure 9). Both B1648 and M41 strains successfully invade and replicate in the epithelium of the respiratory mucosa. B1648 uses more mononuclear cells as carrier cells to breach through the underlying layers of the respiratory mucosa. The penetration through the deeper layers of the respiratory mucosa was more intensive with B1648 compared to M41. B1648 spreads via a cell free and cell-associated viremia to target organs such as kidneys, liver, spleen and lungs whereas M41 does not do this. B1648 showed a fully productive replication in the KUL01^+^ blood monocytic cells. B1648 replication was more productive in kidneys and less in liver, spleen and lungs. During M41 infection, the infected KUL01^+^ cells could not be able to infiltrate into the circulation or were abortive in the submucosal layers of the upper respiratory tract (trachea and bronchi). Our study unveils hitherto unknown, but crucial aspects of the tissue tropism of B1648 and M41 strains. B1648 has achieved highly nephropathogenic hallmarks by exploiting the carrier mononuclear cells in an active manner, whereas M41 is not nephropathogenic due to the abortive nature of M41 infection of mononuclear cells.

## Abbreviations

IBV: infectious bronchitis virus
RT-qPCR: real time quantitative polymerase chain reaction
MERS-CoV: Middle East respiratory syndrome coronavirus
SARS: severe acute respiratory syndrome
SPF: specific pathogen free
EID_50_/mL: 50% egg infectious dose per mL
DPBS: Dulbecco’s phosphate buffered saline
hpi: hours post inoculation
dpi: days post inoculation
ANOVA: one-way analysis of variance
ORF 1a: open reading frame 1a
LDR: linear dynamic range
EHV1: equine herpes virus 1
EAV: equine arteritis virus
BLAST: basic local alignment search tool
URT: upper respiratory tract
BM: basement membrane
RT: room temperature
